# Evaluation of real‐world outcomes associated with use of a prescription digital therapeutic to treat substance use disorders

**DOI:** 10.1111/ajad.13346

**Published:** 2022-10-20

**Authors:** Xiaorui Xiong, Stephen Braun, Maxine Stitzer, Hilary Luderer, Gigi Shafai, Brendan Hare, Michael Stevenson, Yuri Maricich

**Affiliations:** ^1^ Medical Affairs, Pear Therapeutics (US) Boston Massachusetts USA; ^2^ Behavioral Pharmacology Research Unit, Friends Research Institute Baltimore Maryland USA

## Abstract

**Background and Objectives:**

Digital therapeutics can expand the reach and fidelity of behavioral treatment for substance use disorders (SUDs). This analysis evaluated real‐world engagement and clinical outcomes in patients diagnosed with SUD who were prescribed reSET®, an FDA‐authorized prescription digital therapeutic (PDT).

**Methods:**

Patients were prescribed a 12‐week PDT comprising 61 therapy lessons (31 “core” and 30 “keep learning” lessons) and contingency management rewards (positive reinforcement message or monetary gift cards) based on lesson completion and negative urine drug screens. Engagement (defined as any activity in the PDT), retention (any activity in Weeks 9–12), and substance use data were collected automatically by the PDT and analyzed descriptively. Associations between early lesson completion and end‐of‐treatment outcomes were assessed.

**Results:**

Six hundred and fifty‐eight patients filled their prescription. Evaluated were 602 patients who were exposed to therapeutic content by completing at least one lesson (median age 37 years, 33% female, 41% male, 26% unreported sex). Median lessons completed was 33 (out of 61 possible), and 52% of patients completed all core modules. Retention in treatment during the last 4 weeks of treatment was 74%, and 62% were abstinent (missing data considered positive). [Correction added on 13 December 2022, after first online publication: In the preceding sentence, the treatment percentage values were revised from 74.6% to 74%.]

**Discussion and Conclusions:**

Patients with SUD exhibited robust engagement with a PDT, high rates of retention through 12 weeks, and substantial rates of abstinence at end of treatment when the therapeutic was used in a real‐world setting. PDT's hold promise as a new way to access effective SUD treatment.

**Scientific Significance:**

This study is the first to report real‐world PDT engagement and clinical outcomes data from a large, geographically diverse population of patients with SUDs.

## INTRODUCTION

Substance use disorder (SUD), which includes dependence on alcohol, cannabis, stimulants, sedatives, or opioids, affects up to 41 million people in the United States, and yet recent data show that only about 6.5% of these people ever receive SUD treatment.^1^ Evidence‐based behavioral treatments for SUD exist, but even among patients who access SUD treatment, most do not receive these therapies due to a lack of specialty facilities and/or clinicians trained in evidence‐based psychological or behavioral therapies.^2^ Other barriers to widespread use of evidence‐based behavioral approaches include inconsistent delivery, quality, and fidelity across healthcare providers, and high turnover among providers.^3^ These issues are more acute in rural communities, where substance use treatment centers or addiction specialists may not exist or be prohibitively distant.^3^ Means of digitally delivering evidence‐based treatments remotely with fidelity and with minimal clinician involvement may help overcome limitations of existing treatment approaches while improving outcomes.

Patient attrition from SUD treatment is also a common barrier to successful recovery. Roughly a third of those starting an SUD treatment program quit within the first month,^4^ and attrition rates of 50% or higher have been reported within the first 3 months of treatment.^5^ Research has shown a positive association between active engagement in treatment, improved retention, and successful recovery.^6^ Further, there are examples of this association in studies involving digital therapeutics. One randomized trial evaluating a smartphone application for alcohol dependence found that the number of days the application was used and number of pages viewed were significantly associated with a reduction in the number of risky drinking days.^7^ Another study of a computer‐based behavioral intervention for SUD found that the number of sessions attended and number of homework assignments completed were positively associated with abstinence.^8^

Prescription digital therapeutics (PDTs) are software‐based disease treatments that adhere to standards of Good Manufacturing Practices, have been evaluated for safety and efficacy in randomized controlled trials (RCTs), and are authorized by the US Food and Drug Administration (FDA). Prescribed by treating clinicians, and delivered on mobile devices, PDTs may expand access to evidence‐based therapies including those used in the treatment of SUDs. This is especially useful when the SUD involves cocaine, cannabis, and stimulants such as methamphetamines, for which no FDA‐approved pharmacotherapies exist.

The reSET® PDT delivers a 12‐week therapeutic program combining cognitive behavioral therapy (CBT) based on the Community Reinforcement Approach (CRA),^9^ a contingency management (CM) system providing motivational incentives (positive reinforcement messages or monetary gift cards) for lesson completion and abstinence, and fluency training to reinforce concept mastery. Studies have consistently demonstrated that CM interventions, particularly abstinence‐based incentives, can support treatment and recovery in individuals with a wide range of SUDs.^10^

After a clinician prescribes reSET, patients download the therapeutic, enter an access code, and set a password, which allows them to begin working and learning in the PDT. The cost of the PDT is either covered by some form of health insurance or, rarely, by patients themselves.

The PDT content consists of a series of interactive, on‐demand audio, text, and video CBT/CRA lessons that are sequentially unlocked as patients progress through the program. Patients are instructed to complete 4 lessons per week starting with the 31 “core” lessons and then, only when those are completed, an additional 30 supplemental lessons. Core lessons teach basic cognitive‐behavioral and relapse prevention skills and provide education about behavioral risk reduction for infections related to sex or shared needles. Supplemental lessons target improved psychosocial functioning (e.g., managing relationships, building communication skills, and improving time management) and provide in‐depth training on preventing or living with infections.

Most lessons include a fluency training session (e.g., quiz), and upon successful completion of fluency training, the patient can earn CM rewards (either virtual or tangible via digital gift cards redeemable at participating companies) by spinning the rewards wheel.^11^ Patients are eligible for earning CM rewards for completing up to 4 lessons per week or for negative urine drug screens. Prescribing clinicians access patient data and self‐reports via a “clinician dashboard” on a computer or mobile device. No formal training is required for using the clinician dashboard.

The effectiveness of the treatment program available in the PDT was evaluated in several RCTs involving approximately 1500 patients with SUDs. These studies used the Therapeutic Education System, which was the computer‐based precursor product to reSET.^12^, ^13^, ^14^, ^15^, ^16^ The trials demonstrated improved rates of abstinence and treatment retention among patients receiving the computer‐delivered therapy as an adjunct to standard care compared to those who received treatment‐as‐usual alone.

RCTs are the gold standard for evaluating the safety and efficacy of new therapeutics. They are typically conducted with strict inclusion and exclusion criteria and a host of methodologies designed to optimize internal validity and ensure maximum scientific validity. Real‐world evidence, in contrast, often involves heterogeneous patient populations being treated in a diversity of uncontrolled healthcare settings by clincians operating under a range of workflow conditions and with variations in skills and other factors.^17^ Real‐world data (RWD) acquired in the context of typical health care delivery and in the absence of stringent research constraints provides a complementary evaluation of therapeutic performance, including measures of patient engagement and assessments of clinically relevant outcomes.^18^ RWD acquisition informs the generalizability of RCT results and can help inform the implementation of treatment options, the choice of patients, and the setting of patient expectations for treatment outcomes.^19^

This study is the first to report RWD in a large, geographically diverse population of patients with SUDs who were treated with a PDT.

## METHODS

This real‐world observational evaluation included an all‐comer population of patients (i.e., no inclusion/exclusion criteria of any kind) from 28 states who filled an initial prescription for reSET between January 2019 and March 2021. Per the indications for use, reSET is intended for outpatient treatment settings under the supervision of a prescribing clinician. Out of a total of 658 patients who filled a prescription during this time window, 602 patients who completed at least one reSET lesson at any point during their first 12‐week prescription for the therapeutic were included in the main observational analysis as they are considered to have engaged with the primary therapeutic element of the PDT. Since this was an observational analysis with no inclusion or exclusion criteria, no limits were placed on which substance, or substances, were of primary concern for patients, nor on what other pharmacological or behavioral treatments patients might have been using concurrently. All interactions with the PDT, including lesson completion and substance use self‐reports, were automatically uploaded from the PDTs to a central database.

Patients provided permission for use of the data as part of accepting the terms of service for using the PDT, and all patient data were deidentified. The evaluation did not meet the definition of human subjects research as articulated in the Declaration of Helsinki.

Engagement and other therapeutic use data were analysed on a population level with descriptive statistics. Patient engagement was assesed two ways. An “active day” was defined as a day in which a patient used any feature of reSET (i.e., opening the app, viewing a lesson, completing assessments, recording triggers or cravings, or reporting abstinence status). Engagement was also assessed by counting completion of therapeutic lessons during the 12 weeks of treatment.

Substance use was evaluated as a composite of patient self‐reports recorded via the PDT as well as urine drug screens (UDS) performed and recorded by clinicians via the clinician dashboard. As a real‐world evaluation, the data available for these analyses were either entered by the clinicians via the dashboard or by patients' use and data entry into the PDT.

Abstinence was evaluated during the last 4 weeks (Weeks 9–12) of the prescription, an assessment window that is based on the Clinical Trials Network Treatment Effect and Assessment Measures Task Force recommendations to evaluate the effect of a treatment based on abstinence during the last month of treatment^20^ and that has been used in previous trials of this therapeutic.^14^, ^15^ Abstinence during the assessment window was considered a binary outcome, based on available data for each week. An “abstinent week” was defined as a week with no positive self‐reported use or UDS. Consistent with prior real‐world and observational studies,^21^ missing abstinence data for any given week were imputed in two ways. The first considered an individual positive if either any self‐report or UDS was positive in the last 4 weeks or if no data were available (“missing data positive”). This analysis included the entire population of individuals who engaged with the PDT (*n* = 602). The second approach was “missing data excluded” in which patients without any self‐reports or UDS results during any of the last 4 weeks were excluded from the analysis and only patients with either positive or negative reports were included (*n* = 434). To access lessons, patients were required to report drug or alcohol use every 4 days (yes/no; with no requirement to identify specific drugs used) via the PDT. Clinician‐entered negative UDSs were rewarded by giving the patient a chance to earn CM rewards.

Alternatively, to look at substance use data across the entire prescription as a secondary measure, patients were also classified as “responders” defined as having ≥80% of the 12 weeks with negative UDS or self‐reports of no drug use (weeks without data assumed positive for use).

Retention in treatment was defined as any patient activity in the PDT during the last 4 weeks of the prescription. Associations were explored between early engagement with the PDT (i.e., number of lessons completed in Weeks 1–4) and abstinence or treatment retention outcomes in Weeks 9–12.

## RESULTS

This analysis evaluated 602 patients with SUD from 28 states who received their first prescription for reSET, consented to use, filled their prescription, and were exposed to therapeutic content by completing at least one lesson (Table 1). An additional 56 patients filled a prescription but did not complete any lessons. Substances used by patients (as reported by clinicians) were: alcohol (46.7%), opioids (17.9%), stimulants not including cocaine (13.3%), cannabis (7.8%), cocaine (6.5%), and other/unknown (7.8%).

**Table 1:** Patient characteristics [Correction added on 13 December 2022, after first online publication: The second row was revised from “Median age” to “Mean age” and the third row was added.]

Demographics	reSET cohort (*n* = 602)
Mean age at enrollment (years)	39 ± 11.8
Median age at enrollment (years)	37
Age group at enrollment, *n* (%)	
18–29	122 (20.3%)
30–39	223 (37.0%)
40–49	124 (20.6%)
50–59	86 (14.4%)
60+	47 (7.8%)
Gender, *n* (%)	
Female	200 (33.3%)
Male	247 (41.0%)
Not reported	155 (25.7%)

Of the 602 patients, 57% had health insurance through Medicaid, 25% were covered by private health insurance, and 6% were covered by Medicare. Insurance status was not available for 12% of patients.

### Therapeutic use/patient engagement

**Figure 1:**
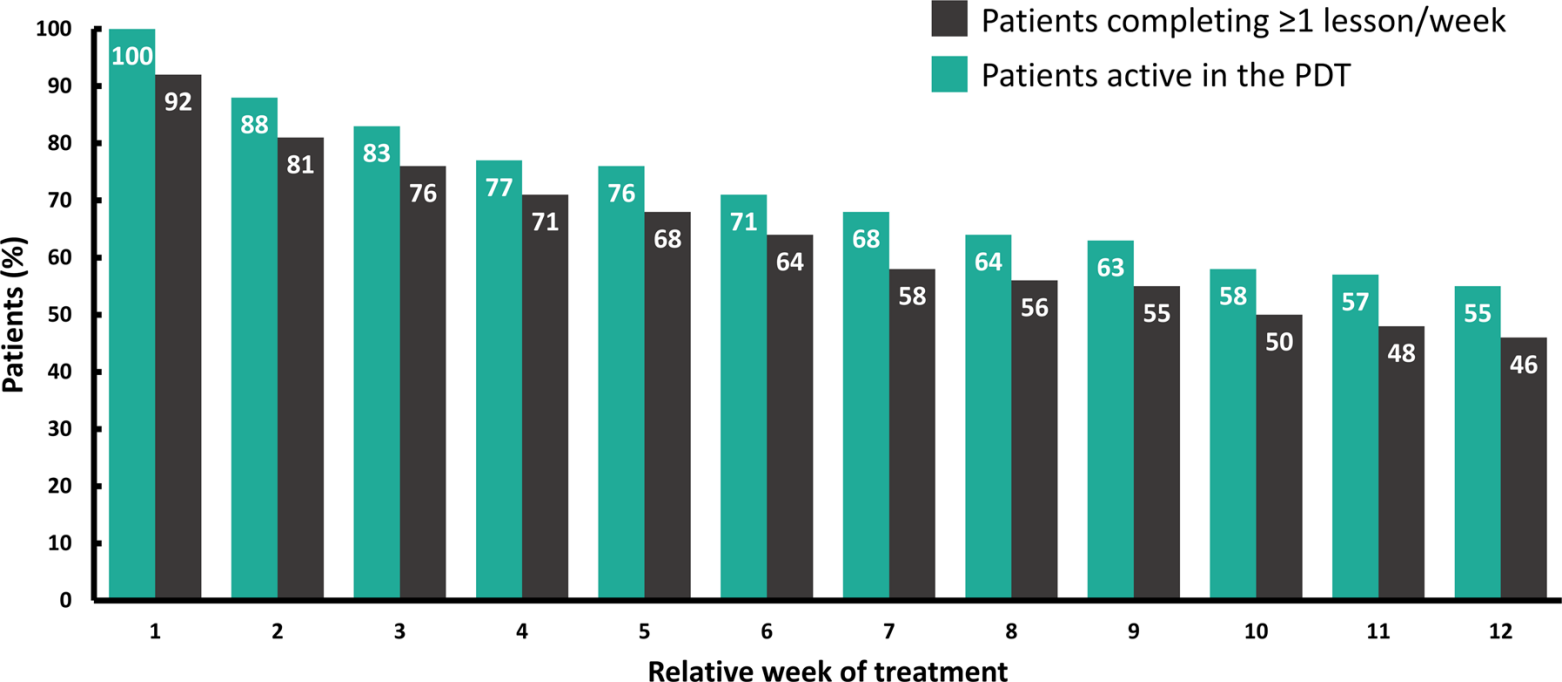
Patterns of PDT engagement over time. Bars show the percentages of patients who either had any type of activity in the PDT (green bars) or who completed at least 1 therapy lesson (black bars) in each of the 12 weeks of treatment (*n* = 602). Actual percent represented is shown within each bar. PDT, prescription digital therapeutic.

As shown in Figure 1 55% of patients were still actively using the PDT in some way during Week 12 while 46% completed one or more therapy lessons that week. The overall median total number of therapy lessons completed during 12 weeks was 33 (interquartile range: 13–39) out of 61 possible.

**Figure 2:**
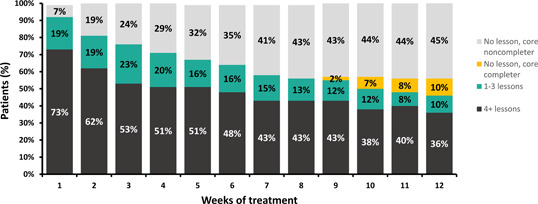
Module completion rate for the evaluated sample (*n* = 602) across the 12‐week PDT treatment duration. Percent completing 0 lessons in a given week are shown in grey or orange, with orange denoting individuals who did not complete any lessons in that week but had previously completed all core lessons. Percent completing between 1 and 3 lessons in a given week are shown in green and those who completed the recommended 4 lessons or more in a given week are shown in black. Actual percentages in each category are shown within each bar. PDT, prescription digital therapeutic.

Figure 2 shows rates of lesson completion by week. The percent of patients completing 4 or more lessons per week declined from 73% in Week 1 to 36% in Week 12. Percent completing any lessons was 92% in Week 1 and 46% in Week 12. By the last 4 weeks of the program some patients with no weekly lesson completion had previously completed all core modules. Within the total sample, 52% completed all core lessons by the end of the prescription. Across Weeks 9–12 of treatment 74% of patients (443 of 602) showed some activity in the PDT and were considered retained in treatment. [Correction added on 13 December 2022, after first online publication: In the preceding sentence, the treatment percentage values were revised from 74.6% to 74%.]

An analysis of PDT usage events by time‐of‐day showed that use occurred throughout the 24‐h period but was greatest and relatively stable between 9 a.m. and 9 p.m. and lowest between midnight and 5 a.m.

The sample included a wide range of age groups: 20% were between 18 and 29 years old, 37% were between 30 and 39, 21% were 40–49, and 22% were 50 years or older. No generational differences in PDT engagement were observed: median total number of lessons completed across the 12‐week prescription was similar across age groups and ranged from 27 to 37 out of 61 possible lessons (27 lessons for those aged 18–29, 36 lessons for 30–39, 32 lessons for 40–49, 28 lessons for 50–59, and 37 lessons for 60+).

### Abstinence

**Figure 3:**
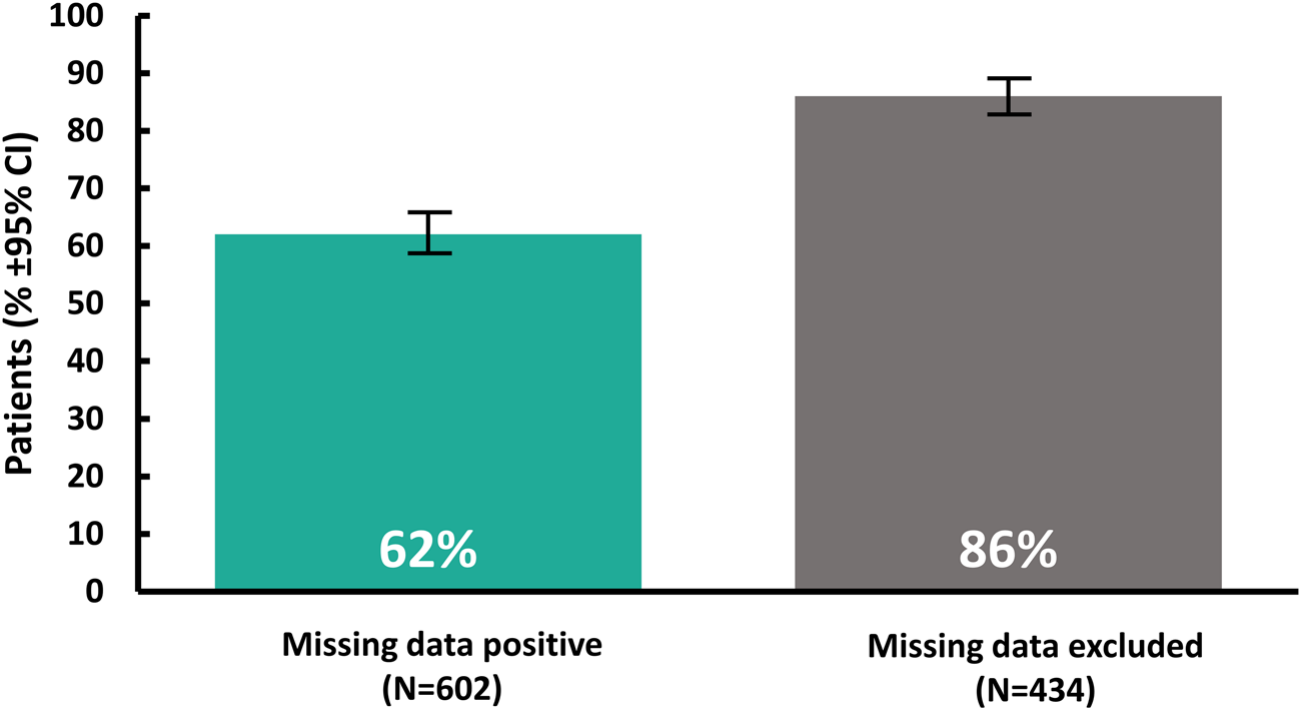
Abstinence in Weeks 9–12 of treatment. Percentage of patients who were abstinent in the last 4 weeks of treatment in an analysis combining self‐report and UDS data using two methods of missing data imputation. “Missing data positive” means that patients who had no abstinence data available in the last 4 weeks were considered nonabstinent (green bar). “Missing data excluded” means that patients who had no abstinence data available in the last 4 weeks (*n* = 168) were excluded from the analysis (black bar).

Four hundred and thirty‐four patients (72%) provided at least one substance use self‐report during Weeks 9–12. A total of 92 patients (15%) had at least one clinician‐entered UDS report during Weeks 9–12. In an analysis combining self‐report and UDS data, abstinence in Weeks 9–12 was 62% using the “missing data positive” analysis (*N* = 602) and 86% were abstinent using the “missing data excluded” (*N* = 434) analysis (Figure 3). Use of the responder criteria identified 40.9% of patients who had an overall abstinence rate ≥80% across the duration of the prescription when missing weeks were assumed to be positive.

### Associations with early engagement

**Figure 4:**
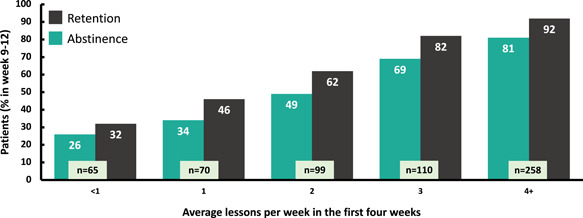
Association between early engagement and end of treatment outcomes. Positive association between early engagement (lessons completed) and end‐of‐treatment outcomes. The average number of modules completed in each of the first 4 weeks of treatment (total lessons/4 and rounded) and the percentage of patients who were either abstinent (green bars) or who were retained in treatment (black bars) in the last 4 weeks of treatment.

Both retention at the end of the prescription period and abstinence were positively associated with the mean number of lessons completed in the first 4 weeks of the prescription (Figure 4). Of 258 patients who completed an average of 4 or more lessons per week in Weeks 1–4, 92% were retained. In contrast, among the 65 patients who completed fewer than 1 lesson per week on average in Weeks 1–4, only 32% were retained. Similarly, abstinence (missing as positive) was 81% among the patients who completed an average 4 or more lessons in Weeks 1–4, but only 26% among the 65 patients who completed an average of fewer than 1 lesson per week in Weeks 1–4.

## DISCUSSION

Patient attrition from SUD treatment is a common barrier to successful recovery, thus treatments or programs that increase engagement with, and retention in treatment may support recovery from SUDs. The RWD from this evaluation strongly suggest that adults of all ages who fill a prescription for a PDT to treat SUD readily engage with the PDT, many at the recommended content dose. Fewer than 10% of those who filled a prescription for the PDT failed to engage with the central therapeutic element of the program, that is, the CBT lessons, while the remainder (92%), who comprised this sample, completed at least one CBT lesson. We observed that 55% of the sample engaged with some feature of the PDT in the last week of the 12‐week treatment period while an even higher number (74%) engaged with the therapeutic at some point during the last 4 weeks of treatment. Early engagement with the PDT, particularly lesson completion, was positively associated in a dose‐dependent manner with both retention and abstinence at the end of treatment, suggesting that patient behavior early in treatment can serve as a prognostic tool and that outcomes may be improved with more intense and personalized focus on early engagement.

Retention rates in SUD treatment are typically low and highly variable and depend on many circumstances including characteristics of the treatment population, philosophy, content, and location of the treatment program. The 74% retention rate (any activity in the PDT in Weeks 9–12) we observed is notable, especially as this evaluation was not a clinical trial, but rather a report of RWD from a broad sample of SUD patients who were assigned the intervention by a clinician and began using it after filling their prescription. These patients were diverse in terms of age, geographic location, and the setting in which they were treated.

The engagement and retention data we observed are consistent with, or higher than, those observed in studies of some other interventions for SUD, including a patient‐centered behavioral intervention (49%),^22^ outpatient drug‐free programs (25%),^6^ and residential SUD treatment programs (48%).^6^

With only 6.5% of patients with SUD receiving any treatment, and high levels of attrition among patients who do receive treatment, a therapeutic that can be conveniently used throughout the day (i.e., outside of typical clinic hours) and is associated with high rates of engagement and retention, offers the potential for overcoming existing limitations in addiction treatment programs.

The abstinence rate of 62% we observed (using a conservative “patients with no data positive” analysis) is encouraging. This rate is higher than has been observed in RCTs such as in the pivotal trials on which FDA approval of reSET^15^ was based, as well as studies of patients treated for other SUDs. For example, in an analysis of 276 SUD treatment follow‐up studies in adult patients, the average abstinence rate across all studies was 47.6%.^23^ This may suggest a degree of self‐selection in the real‐world utilization of PDT's whereby patients who are able to continue using the therapeutic are at more stable levels of recovery. Our responder rate of 40.9% (patients having ≥80% of the 12 weeks with negative UDS or self‐reports of no drug use) is more in line with published literature, although the high rate of missing date combined with the imputation method we used (counting weeks with missing data as positive) means that this rate is a “worst case scenario.”

These positive results on key treatment outcomes may be relevant to clinicians, public health experts, and health care policy makers who are challenged to provide effective, widely‐available treatments to patients with SUDs.

### Limitations

Conducting research in a real‐world patient population coping with the difficult disease of SUD is challenging. Although RWD can enhance generalizability and external validity from RCTs, such evaluations have some intrinsic limitations. First, the absence of comparison groups is a limitation. Second, heterogeneity in patient populations, care settings, and clinician practices exist. Third, the PDT collects only minimal amounts of demographic data (i.e., age, sex) and does not collect information related to the structure and format of treatment programs in which the patient is enrolled, nor about the nature of concurrent pharmaceutical and/or behavioral treatments being received. This limits the ability to examine demographic subgroups or to discern any potential interactions associated with treatment context or receipt of other therapies.

Although data captured by the PDT regarding app utilization and lesson completion is a strength, drug use data input was not systematic and relied on patient self‐reports and clinician entry of urinalysis data. Interpretation is limited by variable and low rates of self‐report and low rates of urinalysis reporting, resulting in heavy reliance on self‐report data. The use in these analyses of a composite measure of abstinence (self‐report and/or UDS), however, is consistent with other RWE and observational studies. Two studies used self‐report/UDS data,^24^, ^25^ and two have used self‐reported abstinence data only.^26^, ^27^

Unlike a clinical trial where UDS and/or self‐report are collected systematically to evaluate effectiveness, decisions to collect UDS in real‐world clinical practice settings are determined by the care model of a particular addiction facility, by clinicians based on the specific needs and status of individual patients, or by attempts to minimize unnecessary testing. For these reasons, the amount and quality of UDS data available for analysis are limited. Missing data is also an issue; data are reported both for samples with and without data available at end of treatment. The responder analysis represents a conservative, worst‐case scenario, imputing missing data as positive, thus is heavily impacted by the decline in available self‐report and UDS data. Interpretation of abstinence rates at end of treatment is limited by lack of pretreatment drug use information and may underestimate or overestimate real rates depending on the case—for example, patients who begin therapy already in remission from drug use have a good prognosis for long‐term abstinence.^28^

## CONCLUSIONS

In this observational evaluation, a large, geographically diverse population of patients with a range of ages and substance use patterns, readily and consistently engaged with the reSET PDT in a real‐world setting and had high rates of retention through 12 weeks. Notably, early consistent engagement with the therapeutic was associated with high rates of abstinence and retention at the end of treatment. These findings suggest the potential benefit of this PDT for treating patients with SUD, a population in need of more effective therapies.

## AUTHOR CONTRIBUTIONS

All authors made a significant contribution to the work reported, whether that is in the conception, evaluation design, execution, acquisition of data, analysis and interpretation, or in all these areas; took part in drafting, revising or critically reviewing the article; gave final approval of the version to be published; have agreed on the journal to which the article has been submitted; and agree to be accountable for all aspects of the work.

## CONFLICTS OF INTEREST

XX, SB, HL, GS, BH, and YM are employees of Pear Therapeutics, Inc. M. Stevenson and M. Stitzer are consultants to Pear Therapeutics (US), Inc.
